# Milk as a Conveyer of Disease1Read before the Passaic County Veterinary Medical Association, at Paterson, N. J., November 4, 1902.

**Published:** 1902-12

**Authors:** T. J. Cooper

**Affiliations:** Paterson, N. J.


### MILK AS A CONVEYER OF DISEASE.[1]

By T. J. Cooper, V.S.,
PATERSON, N. J.

Milk is the original and natural secretion of the mammary glands, similar to the secretions of the salivary glands, and not changed blood as some take it to be.

It is a bluish-white, opaque fluid, with a pleasant, sweet taste.

As these glands are subject to various forms of disease, changing their secretion, the use of milk as food for the human race and animals is of the utmost importance. Necessity demands us to be on the alert to recognize and prevent the spread of the different diseases which are communicated through its use, such as anthrax, foot-and-mouth disease, cholera infantum, typhoid fever, scarlatina, diphtheria, and tuberculosis.

The tracing of anthrax bacillus through milk is not sufficiently proven, though the anthrax bacillus may occur in the milk of animals with the disease, and anthrax does occur in the intestinal form in man.

Foot-and-mouth disease may be caused by milk from affected animals. Hertwig, with two friends, drank the milk from a cow with the disease, and contracted it; Jacob did likewise, with the same result.

In typhoid fever there is a large amount of evidence showing epidemics to be conveyed through the milk. This is strengthened by

[1] Read before the Passaic County Veterinary Medical Association, at Paterson, N. J., November 4, 1902.

the fact that the bacillus typhus thrives in it; the infection was traced to the person carrying the disease from the place where it existed, and milking the cows, also by the dairy pails, which are often cleansed in the kitchen which may open into the sick-room. One case was reported where the infection was carried by the towel used on a patient and afterward used for drying the milk pails.

The epidemics of Svarteborg in 1889 and the one reported by Welpey in 1893 were traced to the modern creamery, which received milk from the neighboring farmers, separated it and returned the skim milk to the contributors. When the milk from any contributing farmer was infected from disease in his family, all the creamery milk received its share of the germs.

In July, 1895, the severest epidemic of typhoid fever of which there is any record in Connecticut, occurred in Stamford. There were 200 cases in that city of 18,000 inhabitants. It was concluded that the disease spread from infected milk, and that it became infected by washing the milk cans with water from a highly polluted well. This shows the necessity of thorough sterilizing the cans and all utensils.

Milk infected with scarlatina can generally be traced to the milkman's home.

Some epidemics of diphtheria have been traced to consumers of a certain supply ; it evidently conveyed the disease.

Cholera infantum has been contracted by drinking milk containing the germs of that disease. Gaffky and Simpson have reported an epidemic caused through milk ; and it is now claimed that this disease is due almost solely to the ingestion of impure milk. Cholera infantum or milk diarrhœa is a disease of infants, and is essentially a preventable disease by the better keeping and caring of the dairy stock, milk, and cleanliness.

Milk as a cause of tuberculosis in the human race and all animals raised on it has caused no small amount of attention among the veterinary and medical professions, as the extent to which it is used is very large, and it can thus spread the disease broadcast.

While practising with Dr. A. Crowforth, of Lockport, N. Y., in 1896 we were called by a farmer to treat a cow with impaction of the rumen. While there he had us examine another cow which he thought had garget ; he had been selling her milk up to a week or so before this. Upon examining her udder we found it very much indurated, no fever present, and the milk very much decreased in quantity. We milked some milk out of each quarter into sterilized bottles, and took them to the laboratory for microscopic examination. The microscopic examination of this milk revealed the presence of a large number of bacilli tuberculosis, which was confirmed by feeding the milk to a three-months'-old kitten, which contracted the disease.

Milk from a tubercular cow, though it does not contain the bacilli tuberculosis, is very injurious to those who consume it if they have the disease ; for, as Prof. James Law first demonstrated, though the milk does not contain the bacilli tuberculosis, it does contain the tuberculin, the toxic properties of the bacilli, and acts on the diseased consumers the same as if they took a small quantity of tuberculin into their systems. It aggravates the existing disease, and produces fever and emaciation, thus hastening the death of the consumer.

Dr. Crowforth fed sixteen kittens on milk from a cow that reacted to the tuberculin test, and the milk proved free from the bacilli tuberculosis by the daily microscopic examination for ten days, followed by the inoculation test in three kittens which proved on post-mortem to be free from the disease.

The remaining thirteen kittens he fed on milk from a cow that did not react to the tuberculin test and did not have the bacilli tuberculosis in the milk, and they all thrived nicely ; then fed them on the milk from a cow that reacted to the tuberculin test and had the bacilli tuberculosis in the milk, and they all contracted the disease and began wasting away, and several died. Then he gave them the milk from the cow that did not react to the tuberculin test and did not have the bacilli tuberculosis in the milk. On this milk they all thrived. He then gave them the milk from the cow that reacted to the tuberculin test but did not have the bacilli tuberculosis in the milk, and they all began to get worse, and presented well-marked symptoms of tuberculosis, and the autopsy on them all showed lesions of the disease.

This experiment thus confirms the opinion that the tuberculin in the milk is as injurious to the affected subjects as the bacilli tuberculosis.

---

Consolidated Fracture of the Second Phalanx Following Neurectomy. Subject, a mare, with articular and tendinous windgalls of both fore fetlocks. After puncturing and injecting antipyrine, tannic acid, and alcohol, and subsequently blistering, median neurectomy was finally performed on the left leg. After some time fracture of the second phalanx occurred and the pieces became united.—*Rev. Vet.*

